# Antinuclear antibody testing in a Turkish pediatrics clinic: is it always necessary?

**DOI:** 10.11604/pamj.2019.32.181.13793

**Published:** 2019-04-11

**Authors:** Erhan Aygün, Fatih Mehmet Kelesoglu, Gafur Dogdu, Aysenur Ersoy, Dilruba Basbug, Dilara Akça, Özge Nur Çam, Berat Akyüz, Tülay Günsay, Ahmet Hakki Kapici, Nur Gökçe Aydin, Edanur Karapinar, Sirin Atay, Nesibe Saglam, Nazli Kübra Okumus, Melike Zeynep Can, Fatmatüzzehra Yazici, Rukiye Eker Ömeroglu

**Affiliations:** 1 Department of Pediatrics, Istanbul Medical School, Istanbul University, Millet Caddesi, Fatih, Istanbul, Turkey; 2 Department of Pediatric Rheumatology, Istanbul Medical School, Istanbul University, Millet Caddesi, Fatih, Istanbul, Turkey; 3 Department of Pediatric Cardiology, Istanbul Medical School, Istanbul University, Millet Caddesi, Fatih, Istanbul, Turkey; 4 Student, Istanbul University, Istanbul Medical Faculty, Millet Caddesi, Fatih, Istanbul, Turkey

**Keywords:** Antinuclear antibody, autoimmune rheumatologic diseases, systemic lupus erythematosus

## Abstract

**Introduction:**

The term anti-nuclear antibody (ANA) is used to define a large group of autoantibodies which specifically bind to nuclear elements. Although healthy individuals may also have ANA positivity, the measurement of ANA is generally used in the diagnosis of autoimmune disorders. However, various studies have shown that ANA testing may be overused, especially in pediatrics clinics. Our aim was to investigate the reasons for antinuclear antibody (ANA) testing in the general pediatrics and pediatric rheumatology clinics of our hospital and to determine whether ANA testing was ordered appropriately by evaluating chief complaints and the ultimate diagnoses of these cases.

**Methods:**

The medical records of pediatric patients in whom ANA testing was performed between January 2014 and June 2016 were retrospectively evaluated. Subjects were grouped according to the indication for ANA testing and ANA titers.

**Results:**

ANA tests were ordered in a total of 409 patients during the study period, with 113 positive ANA results. The ANA test was ordered mostly due to joint pain (50% of the study population). There was an increased likelihood of autoimmune rheumatic diseases (ARDs) with higher ANA titer. The positive predictive value of an ANA test was 16% for any connective tissue disease and 13% for lupus in the pediatric setting.

**Conclusion:**

in the current study, more than one-fourth of the subjects were found to have ANA positivity, while only 15% were ultimately diagnosed with ARDs. Our findings underline the importance of an increased awareness of correct indications for ANA testing.

## Introduction

The presentation of rheumatic diseases in children may be similar to the manifestations of various infections, malignancies and endocrinological disorders. Although laboratory tests have become pivotal in the differential diagnosis of rheumatic diseases, a test which can reliably confirm or exclude rheumatic diseases in children does not exist. In pediatric rheumatology, 80-85% of the data leading to a diagnosis is obtained via a comprehensive medical history. Therefore, obtaining a detailed medical history and meticulous evaluation of the data is of utmost importance in the rheumatology clinic. Medical history should be followed by an extensive physical examination and the clinician should have comprehensive knowledge about rheumatic diseases [1-4]. In addition to clinical evaluation, autoantibody measurements have become a powerful guide for diagnosis and may also provide important data in terms of prognosis, disease activity and treatment of rheumatic diseases. Autoantibody testing has been utilized for the diagnosis and treatment evaluation of autoimmune diseases for more than 50 years [5]. More specifically, antinuclear antibody (ANA) testing has become instrumental in the diagnosis of certain autoimmune rheumatic diseases (ARDs). Quantification of autoantibodies may suggest the presence of an autoimmune disease or inform the clinician about the severity of the disease and/or the immune response associated with the disease [6].

Antinuclear antibodies are a group of autoantibodies which can be detected in systemic autoimmune diseases such as systemic lupus erythematosus (SLE), Sjögren syndrome, systemic sclerosis, inflammatory myositis, mixed connective tissue diseases (MCTD) and rheumatoid arthritis (RA) [7]. However, in the pediatric clinical practice, ANA tests are commonly requested in patients with musculoskeletal complaints, most of which are not related to ARDs. When an ANA test is ordered without strong clinical suspicion for ARDs, there are two outcomes: the result is either negative-and rules out ARDs-or the test is positive, which leads to the requirement for detailed clinical examination and medical history of the patient (which should have been done prior to ANA testing). Ultimately, if the patient is not diagnosed with an ARD, then the test has only caused anxiety for the caretaker of the patient and has increased the number of referrals to pediatric rheumatology clinics. It is important to be aware of the fact that a negative ANA test result is more valuable than a positive one -as it rules out ARDs; however, ANA tests should only be ordered with sufficient clinical suspicion for ARDs. An incomplete understanding of when to request an ANA test and how to interpret the results may reduce patient and caretaker satisfaction and also cause a substantial burden to the healthcare system of a developing country. Thus, evaluating the indications for ANA testing and their results may prove beneficial for the pediatric rheumatology practice and the training of pediatrics residents. In this study, the ANA results of patients who were consulted to pediatric and pediatric rheumatology outpatient clinics with suspicion for autoimmune diseases were reviewed retrospectively. The relationships between chief complaints, final diagnoses and ANA test results and titers were reviewed.

## Methods

In this retrospective single center study, which took place in the general pediatrics and pediatric rheumatology clinic of a university hospital, we reviewed the records of children in whom ANA testing was performed between January 2014 and June 2016. We excluded subjects in which clinical indications for ANA testing were not available. Subjects were grouped according to the indication for ANA testing and ANA titers. The age, gender, chief complaints, ANA test results and final diagnoses of patients were recorded by accessing their data from the hospital information system. The ANA tests were performed by the immunofluorescence technique in microbiology and immunology laboratories. Hep-2 cell lines were used for ANA testing.

**Statistical analysis:** Data analysis was performed with the IBM SPSS v21 software for Windows (IBM Corp. Armonk, NY, USA). We presented categorical data with numbers and percentages and continuous data with means and standard deviations. For the comparison of groups, we used the chi square test for categorical variables and the Student's t test for continuous variables. We considered p-values lower than 0.05 to be statistically significant.

## Results

Antinuclear antibody testing was performed in a total of 409 patients during the indicated study period. The age range of the study population was 5-18 years. We listed reasons for ANA testing requests and study outcomes in Table 1 and the association of ANA titers with ultimate diagnoses in Table 2. Overall, 113 (%27.6) patients had positive ANA test results. ANA test was positive in 15 (%13.2) SLE patients and 18 (%15.9) ARDs. The most common reason for requesting ANA testing was joint pain (50% of the study population). Most of the patients with ANA positivity and ARDs were female. Among ANA positive subjects, girls tended to have a higher rate of ARDs compared with boys, but the difference was not statistically significant (17.7% vs. 8.6%, p > 0.05). None of the patients with ANA titers less than 1:160 were diagnosed with ARDs, while subjects with titers > 1:160 had a similar rate of ARDs (p = 0.2) (Table 3). The positive predictive value of an ANA test was 16% for any connective tissue disease and 13% for SLE. Lupus patients who referred to the clinic with skin and joint symptoms were generally diagnosed as a result of further investigation. Among a total of 64 patients with mucocutaneous symptoms (signs or symptoms involving the hair, skin or oral mucosa), 28 were detected to be ANA positive and 8 of these ANA positive patients were diagnosed with Lupus. Although joint symptoms overlapped with mucocutaneous symptoms in some of the patients, they were evaluated according to their predominant symptom. Patients with joint symptoms constituted 50% of all requests for ANA testing. Although 47 of these patients were diagnosed with JIA and 6 with FMF, the remaining patients with joint symptoms did not demonstrate any specific signs for ARDs. The cause of joint symptoms were considered to be growth pain in many of the remaining subjects. In addition, it was determined that 11 of the patients with widespread pain had vitamin D deficiency.

**Table 1:** Chief complaints of patients in whom antinuclear antibody tests were requested

Chief Complaint	Number of Patientsn=409 (%)
Musculoskeletal disorders (especially joint pain)	207 (%50.6)
Mucocutaneous symptoms (skin, oral and hair problems)	64 (%15.7)
Hematologic disorders	19 (%4.7)
Constitutional symptoms	16 (%3.9)
Abdominal pain	10 (%2.4)
Raynaud's phenomenon	14 (%3.4)
Abnormality in urine urinalysis	8 (%1.9)
Recurrent infections	7 (%1.7)
Other	64 (15.7)

**Table 2:** Characteristics of patients in regard to antinuclear antibody (ANA) results

	ANA positive (n=113)	ANA negative (n=296)	P value
**Age**	10.5	10.1	0.8
**Sex**			
Female	90	157	< 0.001
Male	23	139	
**ARDs**	18	0	< 0.001
Female	16	0	< 0.001
Male	2	0	< 0.001
**Lupus**	15	0	< 0.001
**Polymyositis**	1	0	
**Sjogren**	2	0	

**Table 3:** Antinuclear antibody (ANA) titers

TITER	n (%)	
1/80	13 (%11.5)	
1/160	34 (%30.0)	5 LUPUS, 5 JIA, 2 ITP, 1 PM
1/320	30 (%26.5)	5 LUPUS, 1 SJOGREN, 2 ITP
1/640	19 (%16.9)	2 LUPUS, 3 JIA, 1 ITP
1/1280	16 (%14.2)	2 LUPUS, 2 JIA, 1 SJOGREN
1/2560	1 (%0.9)	1 LUPUS
TOTAL POSITIVE	113(%100)	

Among 50 JIA patients who were tested for ANA, 12 had positive results. Although ANA positivity is associated with uveitis according to the medical literature [8, 9], the evaluation of physical examination records showed that none of our patients had any significant sign of uveitis. Among 13 chronic ITP follow-up patients who had been tested, 5 patients had positive ANA results. Only one of these patients was found to have an ARD. This patient was diagnosed with Sjögren's syndrome in light of antibody test results which were requested with a preliminary diagnosis of autoimmune hepatitis due to liver enzyme elevation. Afterwards, further questioning revealed that the patient had had parotitis attacks which were not recognized by their family. A minor salivary gland biopsy was also consistent with Sjögren's syndrome. Fourteen patients were referred due to Raynaud's phenomenon and 3 were determined to be ANA positive of which one was diagnosed with Lupus. After the capillaroscopic evaluation of the patients who had ANA positive results, various non-ARD abnormalities were determined in 3 patients. Among 8 patients with various urinary system abnormalities such as hematuria and proteinuria, 2 had positive ANA results. However, none of these patients were diagnosed with ARDs with further analysis. One of these patients had been previously diagnosed with idiopathic nephrotic syndrome, but kidney biopsy was ordered due to resistance to corticosteroid treatment and ANA positivity. The biopsy confirmed lupus (full house pattern). Seven patients with recurrent infections were tested for ANA, 2 of them had positive results. None of these patients had an ultimate diagnosis of ARD. Among the 16 patients with constitutional symptoms, only one had ANA positivity. Two of the 16 were diagnosed with FMF and 1 was diagnosed with Kawasaki Disease. Among 10 patients with recurrent abdominal pain, 3 were tested positive for ANA and none were determined to have ARDs.

## Discussion

In pediatrics, unnecessary utilization of ANA testing is very common although the test's specificity and sensitivity are generally low for rheumatic and musculoskeletal system diseases. The ANA test is commonly ordered in patients with musculoskeletal symptoms which are, in most cases, not associated with ARDs. Likewise, the most common cause for requesting ANA in the current study was joint pain (50%). The likelihood of ANA positivity and ARDs tended to be higher in girls compared to boys. The rate of an ARD diagnosis after a positive ANA test was 15% in the current study, and most of these patients were diagnosed with SLE (overall rate: 13%). The overuse of ANA testing is a major problem worldwide. This is partly due to the nature of the test; with titers such as 1:160, the number of false positives are reduced to around 5%, but the possibility for false-negatives increase; the opposite is also true with titers such as 1:40, at which almost 30% of the population are assumed to have a positive result [1-3, 10-13]. Some authors have suggested that positive results at 1:40 titer should be reported in order to identify as many ARD patients as possible [12]. However, this approach increases the number of false-positive results; thus, the clinician should order ANA tests only when there exists a strong suspicion for ARDs and therefore, may confirm or rule-out the diagnosis. A study by Malleson *et al*. showed that, in their center, 41% of ANA tests in children without rheumatic diseases had “positive” results at a titer of 1:20 [14]. This shows the importance of detailed physical examination and thorough medical history prior to ANA testing.

Antinuclear antibody testing should be used as a diagnostic test only when diagnoses of SLE, MCTD and overlap syndromes are considered. In children with signs and symptoms consistent with these ARDs, the ANA test result would almost always be positive [14]. The findings of our study also suggest that, when the signs and symptoms of patients causes the clinician to consider ARDs as probable diagnosis, positive ANA test results can be used to confirm diagnosis. Various studies show ANA positivity to be relatively frequent in the healthy population [14, 15]. Among children, 2-15% have positive ANA, especially with low titers [16, 17]. Therefore, ANA testing should not be used as a screening tool for ARDs in the pediatric setting. However, if it is requested and there is no sign of a systemic disease and the medical history and examination of the child does not suggest ARDs, then positive ANA results in low titers should be considered irrelevant. While ANA positivity has a very high sensitivity for SLE, MCTD and overlap syndromes (as high as 98%), its positive predictive value is very low (10%) [4, 18, 19]. Similar to the literature, we found the positive predictive value of ANA positivity as 13% for SLE in our study. Furthermore, none of the patients with titers lower than 1:160 had an ultimate diagnosis of ARDs. A positive ANA test may indicate the presence of an immune disfunction; however, this situation rarely causes a disease [20]. According to a study performed in a pediatric rheumatology clinic, only 55% of the subjects who had a positive ANA test had an inflammatory rheumatic disease [21]. This rate was relatively lower in our study (28%). However, this may be explained by the inclusion of data from the general pediatrics clinic in addition to the pediatric rheumatology clinic. According to a study in which the clinical use of ANA was investigated, Among 110 subjects with a positive ANA test, 10 had SLE, 18 had JIA, 1 had MCTD, and another patient had Raynaud phenomenon [20]. In our study, 113 patients had positive ANA test results and the distribution of diagnoses were as follows: 15 SLE, 10 JIA, 3 Raynaud phenomenon, 2 Sjogren's syndrome and 1 polymyositis.

Besides the increase in referrals and economical loss caused by the overuse of ANA testing, false-positive results often lead to further follow-up testing, patient/caretaker anxiety, and even misdiagnoses and improper treatments. Narain *et al* [22], in their study comprised of 137 patients with a positive ANA test without a systemic illness, found that 39 had been treated with prednisone at doses as high as 60 mg per day. Raynaud's phenomenon may develop secondarily to SLE, scleroderma and rheumatoid arthritis (RA) in 19% of the patients [23]. This probability increases to 30% if the ANA test is positive and decreases to 7% if the test is negative [24]. Among tthe 14 patients in our study who were referred to the clinic with Raynaud's phenomenon, 3 were determined to be positive for ANAs. In our study, 2 of the 8 patients with hematuria and proteinuria were tested positive for ANA. However, after further analysis, these patients were not diagnosed with any type of ARDs. One of the patients had been previously diagnosed with idiopathic nephrotic syndrome; however, after kidney biopsy-which was ordered due to resistance to corticosteroid treatment and ANA positivity- the patient turned out to have lupus (full house pattern). Another condition where a positive ANA test may be of some value in children is idiopathic thrombocytopenic purpura (ITP). In a study comprised of 87 children with ITP, 36% of those with a positive ANA (titer ≥ 1:40) were found to develop “autoimmune symptoms” [25]. In the current study, 5 of the 13 chronic ITP follow-up patients tested for ANA were found to have ANA positivity.

## Conclusion

More than one-fourth of the subjects included in the study were found to have ANA positivity, while only 15% were ultimately diagnosed with ARDs. We believe that ANA testing may be seen as a screening tool for ARDs by clinicans; while this approach may have merit when a patient has a medical history and examination findings consistent with SLE, MCTD and overlap syndrome, the sensitivity and specificity of the test is too low to be used as a screening test for other ARDs. In addition, false-positive results cause more harm than good for patients and clinicians. Thus, our findings underline the importance of an increased awareness of correct indications for ANA testing in pediatrics clinics.

### What is known about this topic

- ANA positivity to be highly prevalent in both the general and various patient populations;
- ANA tests are overused;
- ANA test is highly specific for some ARDs, such as SLE and Sjögren syndrome, but not for others.

### What this study adds

- No ANA-associated rheumatic disease was identified in patients with an ANA < 1:160;
- According to the information obtained from this study, the ANA test was ordered unnecessarily in many cases;
- These results may be valuable in avoiding unnecessary ordering of the ANA test.

## Competing interests

The authors declare no competing interest.
